# What is the role of aerosol transmission in SARS-Cov-2 Omicron spread in Shanghai?

**DOI:** 10.1186/s12879-022-07876-4

**Published:** 2022-11-24

**Authors:** Gui-Quan Sun, Xia Ma, Zhenzhen Zhang, Quan-Hui Liu, Bai-Lian Li

**Affiliations:** 1 Department of Mathematics, North University of China, Taiyuan, 030051 China; 2 Complex Systems Research Center, Shanxi University, Taiyuan, 030006 China; 3 Department of Science, Taiyuan Institute of Technology, Taiyuan, 030008 China; 4 College of Computer Science, Sichuan University, Chengdu, 610065 China; 5 Department of Botany and Plant Sciences, University of California, Riverside, CA 92521-0124 USA

**Keywords:** Dynamic model, Medical resource, Nucleic acid testing, Asymptomatic case, Vaccination

## Abstract

The Omicron transmission has infected nearly 600,000 people in Shanghai from March 26 to May 31, 2022. Combined with different control measures taken by the government in different periods, a dynamic model was constructed to investigate the impact of medical resources, shelter hospitals and aerosol transmission generated by clustered nucleic acid testing on the spread of Omicron. The parameters of the model were estimated by least square method and MCMC method, and the accuracy of the model was verified by the cumulative number of asymptomatic infected persons and confirmed cases in Shanghai from March 26 to May 31, 2022. The result of numerical simulation demonstrated that the aerosol transmission figured prominently in the transmission of Omicron in Shanghai from March 28 to April 30. Without aerosol transmission, the number of asymptomatic subjects and symptomatic cases would be reduced to 130,000 and 11,730 by May 31, respectively. Without the expansion of shelter hospitals in the second phase, the final size of asymptomatic subjects and symptomatic cases might reach 23.2 million and 4.88 million by May 31, respectively. Our results also revealed that expanded vaccination played a vital role in controlling the spread of Omicron. However, even if the vaccination rate were 100%, the transmission of Omicron should not be completely blocked. Therefore, other control measures should be taken to curb the spread of Omicron, such as widespread antiviral therapies, enhanced testing and strict tracking quarantine measures. This perspective could be utilized as a reference for the transmission and prevention of Omicron in other large cities with a population of 10 million like Shanghai.

## Introduction

Ever since the outbreak of COVID-19 pandemic in December 2019, it has infected more than 500 million people and killed 6 million people worldwide by 02 June, 2022 [1]. The transmission of COVID-19 has brought a huge disaster to the world’s economy and people’s health. During this time, the SARS-CoV-2 has undergone many variations. There are mainly several variants that are harmful and highly infectious to humans, named as Alpha, Beta, Gamma, Delta, Omicron respectively [2]. At present, the most popular mutant strain was Omicron variant which was first discovered in Southern Africa in November 2021 [3]. The Omicron variant of SARS-CoV-2 has fleetly spread globally and superseded Delta variant to be the predominant strain [4]. Compared with the Delta variant, Omicron has presented greater transmissibility and immune escape capability [5–7]. Omicron’s symptoms might be mild due to the protective effects of the vaccine which has been administered worldwide since December 2020. Although Omicron has milder clinical severity relative to Delta[8, 9], the massive infection has devastated healthcare systems around the world, such as US, UK and China[10–12].

**Fig. 1:**
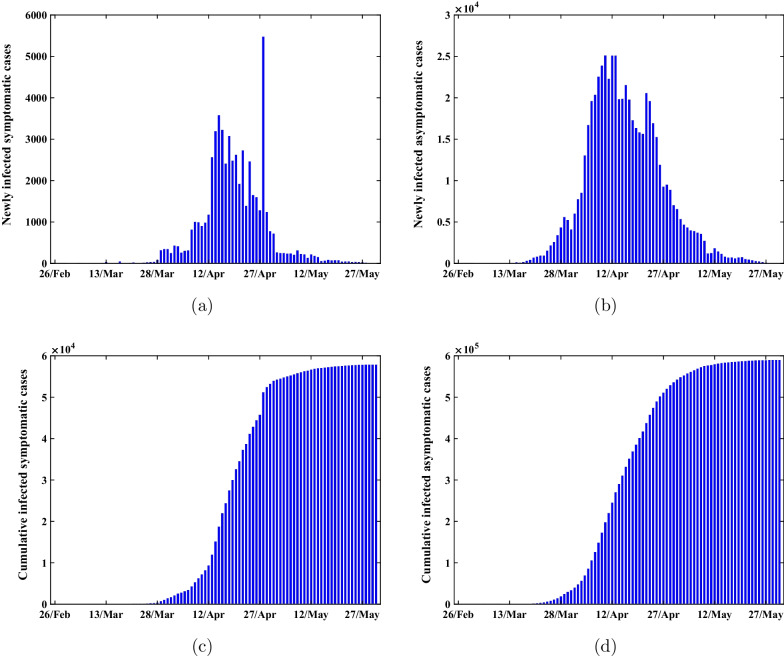
The legally reported data of SARS-CoV-2 variants cases in Shanghai between 26, Feb and 31, May 2022. **a** The Newly infected symptomatic cases. **b** The Newly infected asymptomatic cases. **c** The cumulative infected symptomatic cases. **d**) The cumulative infected asymptomatic cases

Since 2022, Omicron has caused several small outbreaks in many cities in China, such as Tianjin, Anyang and Shenzhen [13, 14]. In addition to massive vaccination, China has adopted a dynamic zero-COVID strategy [15] to cope with SARS-CoV-2 variants and enacted multilayer non-pharmaceutical intervention measures to stem sporadic COVID-19 outbreaks. Excitingly, Omicron has not caused widespread transmission in these areas. However, more than 600,000 local Omicron infections have been reported in Shanghai between 26 February and 31 May, 2022, and about 91% of infections are asymptomatic and the legally reported data of COVID-19 cases in Shanghai during this time are as shown in Figure 1 [16, 17]. Endogenous transmission mechanism of Omicron across Shanghai till remains to be revealed. Understanding the outbreak mechanism of the COVID-19 pandemic has significant meaning for successful containment.

As is well known, the main transmission pathways of COVID-19 in humans is through the inhalation of virus-laden droplets and close contact with infected people [18]. Furthermore, aerosol transmission has been considered to be a supererogatory, yet significant route based on the clinical observations [19, 20]. An aerosol is defined as solid or liquid particles suspended in a gaseous medium between 0.01 and 10 $\upmu$m, which is sufficiently small to enter the human’s respiratory bronchioles and alveoli [21]. Nonetheless, the possibility of aerosol transmission remains controversial, this is partly due to the difficulty of sampling virus-containing aerosols in real-world settings and challenges of quantifying them at low concentrations [22, 23]. Some researchers have intensely hinted that aerosols might be the plausible reason of super transmission on the basis of several confirmed cases in two Wuhan hospitals, China [23], a choir practice in Skagit County, US [24], and a restaurant in Guangzhou, China [25]. Case cluster exposure in hospitals, communities and public transportation have constantly increasing during the epidemic, and aerosol transmission could be generated in some cluster cases when the environmental conditions are appropriate, and it is an indispensable transmission pathway of SARS-CoV-2 [26]. Particularly, a large number of cases were detected during Shanghai’s lockdown in April, 2022. Many experts suspect that aerosol produced by aggregating nucleic acid testing may be responsible for the transmission [27–29]. As such, it is urgent to investigate the epidemiological characteristics of SARS-CoV-2 variants in cluster cases and analyze its transmission routes.

Epidemiological models have been a prominent approach to uncover the characteristics of SARS-CoV-2 transmission, and provide information and guidance for policy-makers to deal with the emerging epidemics [30–42]. Meanwhile, dynamic models could offer valuable insight into the tentative epidemic dynamics [34] and control of SARS-CoV-2 for aerosol transmission [43]. Here, we will leverage a mathematical model to simulate the Omicron wave in Shanghai and excavate the underlying mechanism of the Omicron epidemic. Our research aims to investigate the effect of mitigation measures against SARS-CoV-2 infection through aerosols transmission. Specially, we mainly focus on the role of aerosol transmission on the outbreak dynamics of SARS-CoV-2 in Shanghai, China, as well as the role of vaccination, shelter hospitals, and city lockdown on the epidemic control.

The content of the article is arranged as follows: The research methods and data are presented in the second part. Firstly, according to the transmission characteristics of Omicron in Shanghai, a generalized compartment SEIR model was established. Secondly, the parameters of the model were estimated by least square method and MCMC method, and the accuracy of the model was verified by the cumulative number of asymptomatic infected persons and confirmed cases published by Shanghai Municipal Center for Disease Control and Prevention and Shanghai Municipal Health Commission [16, 17]. The main results are demonstrated in the third part, including the comparison between model predicted value and the actual data, and the impact of different control measures, such as lockdown, aerosols transmission, shelter hospitals, medical resources and vaccine coverage, on the spread of Omicron in Shanghai. The last part is the conclusion.

## Methods and survey data

### Modelling the SARS-CoV-2 transmission with vaccination and aerosols

**Fig. 2:**
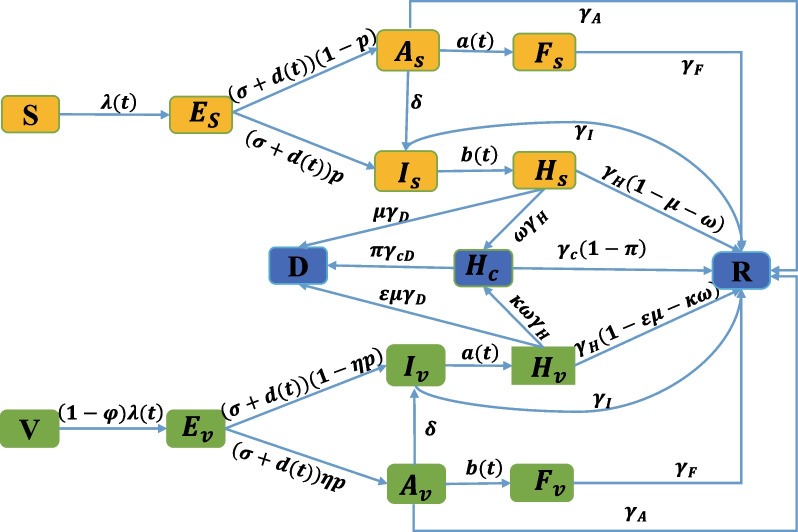
Transmission diagram of Omicron in Shanghai. The yellow compartments represent the infection process of the unvaccinated, and the green compartments represent the infection process after vaccination. Therein, the force of infection for a susceptible individual is approximated as $\lambda (t)=-(\beta (t)+f\xi )\dfrac{\theta (A_s+A_v)+I_s+I_v}{S+V+E_s+E_v+A_s+A_v+I_s+I_v}$

In this part, we will construct a compartmental susceptible-exposed-infectious-removed (SEIR) model (see Fig. 2) to simulate the infection risk from aerosol transmission of Omicron variant in Shanghai, China. Combined with the actual transmission characteristics of Omicron in Shanghai, the total population is divided into 15 compartments at time *t*: the unvaccinated susceptible population *S*(*t*), vaccinated susceptible population *V*(*t*), latent unvaccinated population $E_s(t)$, latent vaccinated population $E_v(t)$, infectious unvaccinated asymptomatic cases $A_s(t)$, infectious vaccinated asymptomatic cases $A_v(t)$, infectious unvaccinated symptomatic cases $I_s(t)$, infectious vaccinated symptomatic cases $I_v(t)$, unvaccinated patients in shelter hospital $F_s(t)$, vaccinated patients in shelter hospital $F_v(t)$, unvaccinated patients in designated hospital $H_s(t)$, vaccinated patients in designated hospital $H_v(t)$, patients in the ICU $H_c(t)$, death population who infected the Omicron *D*(*t*), and recovered population *R*(*t*), respectively. Before constructing the dynamic model, we first make some assumptions as follows:

1. We suppose that susceptible individuals can be infected through contacting with asymptomatic and symptomatic cases, as well as aerosols accumulated during nucleic acid testing. The force of infection for a susceptible individual is approximated as $\lambda (t)$. We utilized the standard incidence, $\lambda (t)=-(\beta (t)+f\xi )\dfrac{\theta (A_s+A_v)+I_s+I_v}{S+V+E_s+E_v+A_s+A_v+I_s+I_v}$, which is assumed that the individuals not in the shelter hospitals and designated hospitals are homogeneously mixed. $\beta (t)$ is the transmission rate by close contact with infectious, *f* is the transmission rate by aerosol, $\xi$ is the probability of exposure to aerosol, $\theta$ is the infectivity of asymptomatic cases relative to symptomatic cases.
2. The transmission rate $\beta (t)$ is subject to the time, non-pharmaceutical interventions and corresponding policy changes. Due to the limitation of medical resources, the nucleic acid detection rate *d*(*t*), the average transfer rate *a*(*t*) from asymptomatic onset to shelter hospital, and average transfer rate *b*(*t*) from symptom onset to designated hospitals are ruled by the time, policy and medical resources.
3. It is assumed that asymptomatic infected people will be quarantined in shelter hospitals, and symptomatic infected people are treated in designated hospitals. When infected people enter the shelter hospitals and designated hospitals, they no longer pose transmission risk to people in society.
4. Vaccination does not provide 100% protection against Omicron variant, but it can reduce the risk of infection, hospitalization and death [40].

Based on the hypotheses above, the transmission diagram is shown in Fig. 2 and the parameters are described in Table 1. The corresponding compartmental model is as follows:

**Table 1:** Definitions of frequently used parameters

Variables	Description
$\beta (t)$	Transmission rate by close contact with infectious
*f*	Transmission rate by aerosol
$\xi$	Probability of exposure to aerosol
$\theta$	Infectivity of asymptomatic cases
	relative to symptomatic cases
$\varphi$	Reduction of transmission rate due to vaccination
$1/\sigma$	Average duration of latent period
*d*(*t*)	Detection rate of nucleic acid testing
*p*	Proportion of an unvaccinated latent who developed symptoms
$\eta$	Proportion of a vaccinated latent who developed
	symptoms relative to an unvaccinated latent
$1/\gamma _A$	Average infectious periods of asymptomatic population
*a*(*t*)	Average transfer rate from asymptomatic onset to shelter hospital
$\delta$	Transfer rate from asymptomatic cases to symptomatic cases
$1/\gamma _I$	Average infectious periods of symptomatic population
	hospitalizations relative to an unvaccinated infection
*b*(*t*)	Average transfer rate from symptom onset to designated hospital
$1/\gamma _F$	Length of shelter hospital stay before recovery
$1/\gamma _H$	Length of hospital stay before recovery
$\mu$	Fatality rate among unvaccinated hospitalized patients
$\omega$	Proportion of unvaccinated hospitalized patients requiring ICU
$\varepsilon$	Proportion of fatality rate among vaccinated hospitalized patients
	relative to an unvaccinated patients
$\kappa$	Proportion of vaccinated hospitalized patients requiring
	ICU relative to an unvaccinated patients
$\pi$	Fatality rate among ICU patients
$1/\gamma _c$	Length of ICU stay before recovery death
$1/\gamma _{cD}$	Average time from ICU admission to death
$1/\gamma _D$	Average time from hospital admission to death

1 $$\begin{aligned} \left\{ \begin{array}{l} \dfrac{dS(t)}{dt}=-[\beta (t)+f\xi ]\dfrac{S[\theta (A_s+A_v)+I_s+I_v]}{S+V+E_s+E_v+A_s+A_v+I_s+I_v},\\ \dfrac{dV(t)}{dt}=-[\beta (t)+f\xi ](1-\varphi )\dfrac{V[\theta (A_s+A_v)+I_s+I_v]}{S+V+E_s+E_v+A_s+A_v+I_s+I_v},\\ \dfrac{dE_s(t)}{dt}=[\beta (t)+f\xi ]\dfrac{S[\theta (A_s+A_v)+I_s+I_v]}{S+V+E_s+E_v+A_s+A_v+I_s+I_v}-\sigma E_s-d(t)E_s,\\ \dfrac{dE_v(t)}{dt}=(\beta (t)+f\xi )(1-\varphi )\dfrac{V[\theta (A_s+A_v)+I_s+I_v]}{S+V+E_s+E_v+A_s+A_v+I_s+I_v}-\sigma E_v-d(t)E_v,\\ \dfrac{dA_s(t)}{dt}=[\sigma +d(t)](1-p)E_s-\gamma _A A_s-a(t)A_s-\delta A_s,\\ \dfrac{dA_v(t)}{dt}=[\sigma +d(t)])(1-\eta p)E_v-\gamma _A A_v-a(t)A_v-\delta A_v,\\ \dfrac{dI_s(t)}{dt}=[\sigma +d(t)] p E_s-\gamma _I I_s-b(t)I_s+\delta A_s,\\ \dfrac{dI_v(t)}{dt}=[\sigma +d(t)]\eta p E_v-\gamma _I I_v-b(t)I_v+\delta A_v,\\ \dfrac{dF_s(t)}{dt}=a(t)A_s-\gamma _FF_s,\\ \dfrac{dF_v(t)}{dt}=a(t)A_v-\gamma _FF_v,\\ \dfrac{dH_s(t)}{dt}=b(t)I_s-\gamma _H(1-\mu -\omega )H_s-\omega \gamma _HH_s-\mu \gamma _DH_s,\\ \dfrac{dH_v(t)}{dt}=b(t)I_v-\gamma _H(1-\varepsilon \mu -\kappa \omega )H_v-\kappa \omega \gamma _HH_v-\varepsilon \mu \gamma _DH_v,\\ \dfrac{dH_{c}(t)}{dt}=\omega \gamma _HH_s+\kappa \omega \gamma _HH_v-\gamma _{c}(1-\pi )H_c-\pi \gamma _{cD}H_c,\\ \dfrac{dD(t)}{dt}=\mu \gamma _DH_s+\varepsilon \mu \gamma _DH_v+\pi \gamma _{cD}H_c,\\ \dfrac{dR(t)}{dt}=\gamma _A A_s+\gamma _A A_v+\gamma _I I_s+\gamma _I I_v+\gamma _FF_s+\gamma _FF_v\\ +\gamma _H(1-\mu -\omega )H_s+\gamma _H(1-\varepsilon \mu -\kappa \omega )H_v+\gamma _{c}(1-\pi )H_c.\\ \end{array} \right. \end{aligned}$$

### Survey data and parameter estimation

The modeling research and simulation just depend upon the publicly available aggregated data. Shanghai Municipal Center for Disease Control and Prevention (SCDC) and Shanghai Municipal Health Commission are authoritative organizations for the prevention and control of epidemic in Shanghai, China [16, 17]. Through the websites and platforms, we can collect the data of daily asymptomatic subjects, daily symptomatic cases, daily asymptomatic subjects turning to confirmed cases, daily death cases, hospitalized cases. The population size used in the simulation are collected from the Shanghai Statistics Bureau, and the information about vaccination data comes from reference [40].

We collected the daily number of asymptomatic infected persons and confirmed cases in Shanghai from February 26 to May 31, 2022 from Shanghai Municipal Health Commission [16]. More than 600, 000 local Omicron infections have been reported in Shanghai, China. According to the changes of epidemic prevention and control measures taken by Shanghai government during this period, the spread of COVID-19 can be divided into three stages. The first stage was the pre-lockdown stage from February 26 to March 28, 2022. During this stage, we assumed that it was free transmission stage without Non-pharmaceutical interventions. Due to small number of infectious cases, nucleic acid testing was not conducted for all people, and aerosol transmission generated by nucleic acid testing was not considered. With the outbreak epidemic of Omicron, Shanghai government began to take lockdown measures gradually from March 28, 2022. The second stage was from March 28 to April 30, 2022. During this period, Shanghai was almost under lockdown, and everyone was practically housebound except some medical workers and those providing supplies. As the number of infectives increased dramatically, medical resources became extremely scarce and then slowly eased. The third stage was from May 01 to May 31, 2022. This stage began to be gradually unsealed and people in low-risk areas were allowed to move freely within the area. The number of beds in shelter and designated hospitals was relatively sufficient. Medical resources have been balanced, and patients in need of hospitalization could be treated in a timely and effective manner. Hereafter, the model (1) will be utilized to simulate the cumulative number of asymptomatic persons and symptomatic cases from February 26 to May 31, 2022. We introduce that *X*(*t*) and *Y*(*t*) represent the cumulative number of asymptomatic persons and symptomatic cases, respectively.

$$\begin{aligned} \dfrac{dX(t)}{dt}=(\sigma +d(t))[(1-p)E_s+(1-p\eta )E_v], \dfrac{dY(t)}{dt}=(\sigma +d(t))(pE_s+p\eta E_v). \end{aligned}$$

Some model parameters (including the transmission rate, probability of exposure to aerosol, detection rate of nucleic acid testing, average transfer rate from asymptomatic onset to shelter hospital, average transfer rate from symptomatic onset to designated hospital), and initial values of the disease states can be estimated by a nonlinear least-squares method. And then we will calibrate the model based on the data on cumulative asymptomatic persons and symptomatic cases. The mean incubation period time of Omicron is assumed to be 1.52 days ($1/\sigma =1.52$) according to references [40, 41]. Studies have shown that in actively ventilated choirs, the probability of aerosol transmission without masks was 0.73 [44]. Since people take off their masks and open their mouths for nucleic acid tests, we assume that the probability of concentrated aerosol transmission due to clustered nucleic acid detection is about 73%. When the medical resources are normal, the average transfer time from confirmed cases to hospitalization is 2.2 days [45], namely $b=1/2.2$. Currently, it is believed that the infectivity of asymptomatic population relative to symptomatic population $\theta$ is 0.75 according to the COVID-19 pandemic [46]. Since Omicron may evade vaccines immunity of neutralizing antibodies resulting in a decrease in the effectiveness of existing vaccines against the Omicron [47, 48]. Studies showed that the Pfizer vaccine effectiveness against infection decreased from 80% for Delta to 33% for Omicron [49]. In addition, vaccines are not completely effective, so we assume that the reduction of transmission rate due to vaccination $\varphi$ is 0.15. Other parameters can be given in Table 2.

**Table 2:** Values of frequently used parameters

Variables	Values	Source	Variables	Values	Source
*f*	0.73	[44]	$\mu$	0.00654	[40]
$\theta$	0.75	[46]	$\omega$	0.019	[40]
$\varphi$	0.15	Assumed	$\varepsilon$	0.3	[40]
$1/\sigma$	1/1.52	[40, 41]	$\kappa$	0.3	[52]
$\eta$	0.5	Assumed	$\omega$	0.019	[40]
$1/\gamma _A$	1/5.64	[50]	$\pi$	0.233	[53]
$1/\gamma _I$	1/10	Assumed	$1/\gamma _c$	1/10	Assumed
$1/\gamma _F$	1/7	[40, 51]	$1/\gamma _{cD}$	1/10	Assumed
$1/\gamma _H$	1/8	[40, 51]	$1/\gamma _D$	1/10	Assumed

The total population size in Shanghai is 24,871,100 and the average vaccination rate for COVID-19 vaccine is 72.6% [40]. We take the initial values of the disease state variables as $S(0)=6806881,~V(0)=18064219,~I_s(0)=0,~ I_v(0)=I_s(0)=0,~F_s (0)=F_v(0)=0,~H_s=H_v=0,~D=R=0$. $E_s(0),~E_v(0),~A_s(0), A_v(0)$ will be estimated by the data fitting. We will calibrate the model by the cumulative asymptomatic persons and symptomatic cases of Omicron from February 26 to May 31, 2022 in Shanghai based on nonlinear least-squares method.

**Fig. 3:**
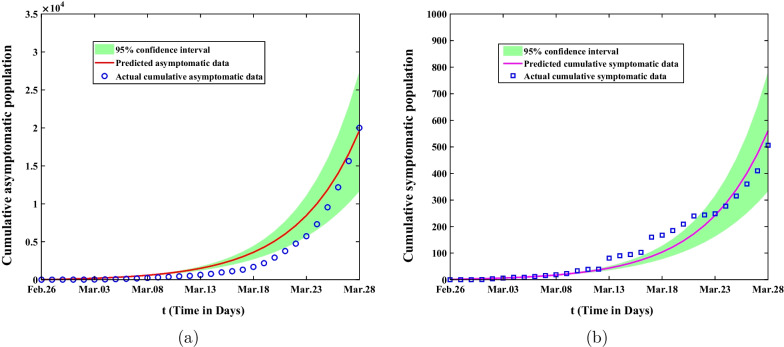
The fitting result of cumulative asymptomatic population and symptomatic population from February 26 to March 28, 2022. The blue circles represent the actual cumulative asymptomatic subjects. The blue squares show the actual cumulative symptomatic cases. The red curve in **a** demonstrates the predicted cumulative asymptomatic data and the purple curve in **b** depicts the predicted cumulative symptomatic data. The green shaded areas show 95% confidence intervals of actual values. **a** The fitting result of cumulative asymptomatic population; **b** The fitting result of cumulative symptomatic population

In the first stage, we considered the spread of Omicron in Shanghai as normal free transmission without aerosol transmission and non-pharmaceutical interventions, $\xi =0$ and $\beta (t)$ were assumed to be constant. Then the remaining parameters need to be estimated were *d*(*t*),  *a*(*t*). Due to small number of infectious cases, we supposed that the detection rate of nucleic acid testing *d*(*t*) and the average transfer rate from asymptomatic to shelter hospital *a*(*t*) were all constants. We could calculate that $p=0.04,~\delta =0.0009$ [16, 17, 54]. In addition to $\beta$, *d* and *a*, other parameters are calculated and given in Table 2. Here, we will utilize the nonlinear least square method and Markov-Chain Monte-Carlo (MCMC) simulations to estimate the parameter values of $\beta$ , *d*, *a* based on the adaptive combination Delayed Rejection and Adaptive Metropolis (DRAM) algorithm [55, 56]. MCMC method is mainly made up of M-H algorithm and Gibbs sampling. Gibbs sampling is realized by the Latin Hypercube Sampling [57] which is comprehensively applied in statistics to assess isochronous variation of numerous parameters, and the adaptive Metropolis-Hastings algorithm. The grundgedanke is to construct a stationary distribution as the Markov chain of the posterior distribution through repeated sampling, thus acquiring the joint posterior distribution samples. Afterwards, we can execute all sorts of statistical inferences according to these samples. Running the algorithm for 10,000 iterations with a burn-in of first 8,000 iterations, the convergence of chains can be evaluated by the Geweke convergence method. The parameter estimation results are showed in Table 3 and the fitting results of cumulative asymptomatic cases and symptomatic cases are shown in Fig. 3.

**Table 3:** Parameter estimation for $\beta$, *d* and *a* with the method of MCMC

Parameter	Mean	Standard	MC error	Geweke
$\beta$	1.613	0.11382	0.004307	0.9959
*d*	0.5007	0.07611	0.002761	0.9371
*a*	0.618	0.07279	0.002847	0.9864

In the second stage from March 28 to April 30, 2022, Shanghai was basically in lockdown and medical resources were relatively tight. Under this situation, as the number of infectives increased, the government started to call for people to stay at home and nucleic acid test was carried out for all people. Faced with the huge number of asymptomatic persons, medical resources began to become strained, and the number of beds in shelter hospitals was seriously insufficient. The government set about transforming and expanding shelter hospitals and designated hospitals. Through the unremitting efforts of the country and government, the shortage of medical resources has gradually eased. On April 30, the number of beds in makeshift hospitals and designated hospitals reached a tight balance [58]. During this period, the parameters greatly affected by policy changes were $\beta (t),~\xi ,~d(t),~a(t),~b(t)$. The transmission rate $\beta (t)$ might vary in response to the various non-drug intervention strategies and political actions. Inspired by reference [37, 38], we assumed $\beta (t)$ to be a hyperbolic tangent type ansatz,

$$\begin{aligned} \beta (t)=\beta _0+\frac{1}{2}\Big [1+\tanh \Big (\frac{t-t^*}{T}\Big )\Big ](\beta _0-\beta ^*), \end{aligned}$$

where $\beta _0$ is the transmission rate as in the first stage, $\beta ^*$ is the minimum transmission rate under the various Non-pharmaceutical interventions and assumed to be 0.1, $t^*$ is the adaptation time, and *T* is the transition time. Due to the large number of people waiting to be tested and the shortage of medical resources, the rate of nucleic acid testing presented a decreasing form. The nucleic acid detection rate *d*(*t*) was presumed to be a decreasing Logistic function, $d(t)=d_0+\dfrac{d_m-d_0}{1+e^{-r_0 (t-t_0)}}$, where $d_0$ is the baseline nucleic acid detection rate, $d_m$ is the minimum nucleic acid detection rate during the outbreak, $r_0$ is the change rate, $t_0$ is the adaptation time. We can calculate that in the second stage $p=0.108$ based on the data [16, 17]. As the number of confirmed infections has soared, medical workers and beds numbers in shelter and designated hospitals were insufficient and the transfer rate of infective people showed a decreasing trend. With the expansion of shelter and designated hospitals and the assistance of medical staff from other provinces, the shortage of medical resources has gradually eased. Given this phenomenon, we supposed that *a*(*t*) and *b*(*t*) were quadratic functions which decreased first and then increased. According to the news reports on April 13 [59], a section titled “ Shanghai Xuhui District Yongkang Street citizens call for help” spread on the Internet, affected the hearts of citizens and netizens. The caller was a resident Mr Yu, who had been diagnosed a week before he was arranged for hospitalization due to the lack of beds in designed hospitals. As such, we could suppose that the transfer rate *b*(*t*) reached a minimum value $\frac{1}{7}$ on April 13 and then gradually increased to $\frac{1}{2.2}$ on April 30. Analogously, we assumed that *a*(*t*) reached a minimum of $\frac{1}{5}$ on April 13 and then gradually increased to normal on April 30. Utilizing the cftool function of Matlab for data fitting could be obtained that $b(t)=0.001172x^2-0.04101x+0.4944,~a(t)=0.001128x^2-0.03947x+0.5383$. At this stage, the government required all people to take nucleic acid tests in fixed testing sites, which was bound to cause aggregation. In addition, nucleic acid testing required the removal of masks and mouth opening, which could form aerosol transmission. The parameters to be estimated here were $\xi ,~T,~d_m,~r_0,~t_0,~t^*,~\delta$, and we used the similar method for the parameter estimation as in the first stage and the results of parameter estimation are shown in Table 4.

**Table 4:** Parameter estimation for $\xi$, *T*, $d_m$, $r_0$, $t_0$, $t^*$ and $\delta$ with the method of MCMC

Parameter	Mean	Standard	MC error	Geweke
$\xi$	0.27159	0.01769	0.001099	0.9782
*T*	14.177	0.1702	0.010573	0.9956
$d_m$	0.37882	0.04292	0.002541	0.9928
$r_0$	0.49833	0.05778	0.004803	0.9899
$t_0$	12.8	1.4174	0.079586	0.9924
$t^*$	10.375	0.31448	0.019102	0.9897
$\delta$	0.01	0.00118	6.885e-05	0.9873

In the third stage from May 01 to May 31, 2022, Shanghai was in a phase of gradual deregulation, and people in low-risk areas could move freely in regulated area. Online shopping, community group buying, take-aways and supplies of daily life gradually returned to normal. As people’s contact gradually increased, and the risk of contact transmission might magnify. With the sufficient amount of social volunteer workers and the improvement of work efficiency, door-to-door nucleic acid services were carried out for the risk groups. The personnel at the nucleic acid testing points queued up in an orderly manner, and the phenomenon of clustering rarely occurred, and the probability of aerosol transmission started to decrease. Following the availability of medical personnel and an increase in the number of nucleic acid sampling sites, nucleic acid testing rates began to increase relative to the second phase. Due to the number of infected people decreased, the number of beds in designated and makeshift hospitals were sufficient. As a consequence, we could presume that the transfer rates of hospitalization and isolation rate of infected people were the same as those in the first stage. We could calculate that $p=0.086$ in the third stage based on the data from [16, 17]. During this stage, the parameters to be estimated were $\xi ,~\beta (t),~d(t),~\delta$ and all parameters were assumed to be constants. we continue to utilize the nonlinear least square and MCMC methods to estimate the parameters, and the results of parameter estimation are shown in Table 5.

**Table 5:** Parameter estimation for $\beta$, $\xi$, *d* and $\delta$ with the method of MCMC

Parameter	Mean	Standard	MC error	Geweke
$\beta$	0.7235	0.0436	0.00183	0.9947
$\xi$	0.1999	0.0573	0.00217	0.9874
*d*	1.0981	0.077	0.00402	0.9969
$\delta$	0.0569	0.0031	0.000233	0.9915

## Results

### Fitting results of Omicron transmission in Shanghai

**Fig. 4:**
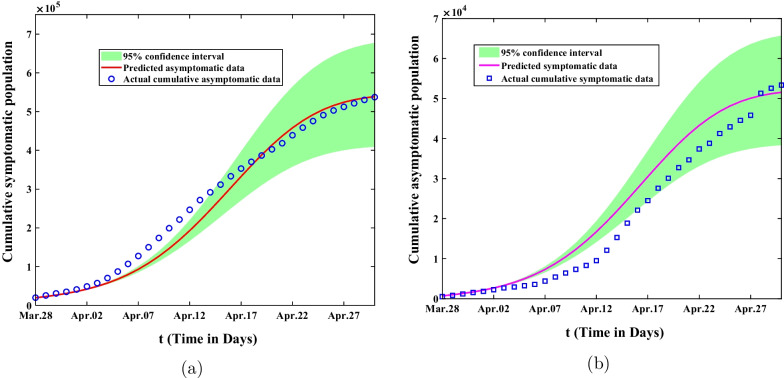
The fitting result of cumulative asymptomatic population and symptomatic population from March 28 to April 30, 2022. The blue circles represent the actual cumulative asymptomatic cases and the blue squares show the actual cumulative symptomatic cases. The red curves demonstrate the predicted cumulative asymptomatic cases and purple curves demonstrate the predicted cumulative symptomatic cases of the model. The green shaded areas show 95% confidence intervals of predicted values. **a** The fitting result of cumulative asymptomatic population. **b** The fitting result of cumulative symptomatic population

**Fig. 5:**
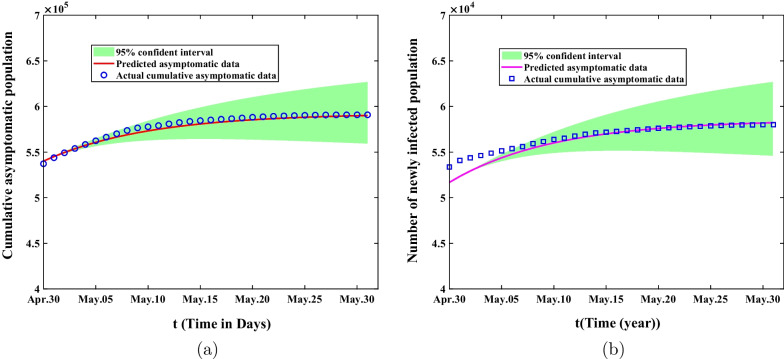
The fitting result of cumulative asymptomatic population and symptomatic population from April 30 to May 31, 2022. The blue circles represent the actual cumulative asymptomatic cases and blue squares show the actual cumulative symptomatic cases. The red curves demonstrate the predicted cumulative asymptomatic cases and the purple curves express the predicted cumulative symptomatic cases of the model. The green shaded areas show 95% confidence intervals of predicted values. **a** The fitting result of cumulative asymptomatic population. **b** The fitting result of cumulative symptomatic population

In the first stage from February 26 to March 28, 2022, the mean value, standard deviation, MC error and Geweke values of estimated parameters were showed in Table 2. The comparison of predicted cumulative asymptomatic persons and symptomatic cases of model (1) with actual values were shown in Fig. 3. The blue circles represented the actual cumulative asymptomatic cases and the blue squares showed the actual cumulative asymptomatic persons. The red curve in Fig. 3a demonstrated the predicted cumulative asymptomatic data of the model and the purple curve in Fig. 3b showed the predicted cumulative symptomatic data. The green shaded areas showed 95% confidence intervals of mean predicted values of model (1). Although the predicted values of the model differed from the actual values at the beginning time, they would eventually fall within the 95% confidence interval of the predicted values. In the second stage from March 28 to April 30, 2022, we could obtain that the mean value, standard deviation, MC error and Geweke values of estimated parameters are exhibited in Table 3. The comparison of predicted cumulative asymptomatic individuals and symptomatic cases of model (1) with actual values were shown in Fig. 4. The predicted and actual numbers of confirmed cases were slightly different but they all ended up within 95% confidence interval of the predicted values. The spike of symptomatic cases on April 27 was due to the fact that many asymptomatic subjects were turned into confirmed cases on that day and then resulting in the discrepancy between the predicted confirmed cases and the actual cases. In the third stage from May 01 to May 31, 2022, we achieved the fitting results about the comparison of predicted cumulative asymptomatic subjects and symptomatic cases of model (1) with actual values in Fig. 5. It could be found that the cumulative infected asymptomatic subjects and symptomatic cases predicted by the model were nearly agreement with the actual reported cases.

### The spread of Omicron without lockdown in Shanghai

**Fig. 6:**
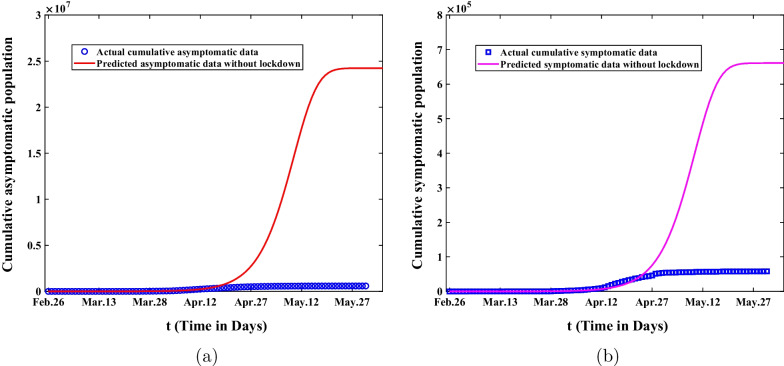
The comparisons of predicted value and actual data about the cumulative asymptomatic population and symptomatic population are showed without the lockdown throughout Shanghai. **a** The predicted cumulative asymptomatic population without lockdown. **b** The predicted cumulative asymptomatic population without lockdown

In the second stage from March 28 to April 30, 2022, if the lockdown was not adopt in Shanghai and the government did not take strict control measures, the virus would continue to spread as in the first stage. The simulation results of cumulative asymptomatic and symptomatic infected persons were shown in Fig. 6. Figure 6a showed the comparison of predicted cumulative asymptomatic subjects with actual cumulative asymptomatic values. We could observe that there would be more than 24 million asymptomatic infected persons on May 31, the final size of asymptomatic infections would increase forty times. Figure 6b demonstrated the comparison of predicted cumulative symptomatic value with reported cumulative symptomatic data. It wasn’t hard to see that it would eventually result in 660,000 symptomatic cases and the final size of symptomatic infections would increase ten times. It also illustrated that it was extremely necessary for the government to adopt lockdown and strict non-drug intervention measures in Shanghai.

### The spread of Omicron without aerosol transmission

**Fig. 7:**
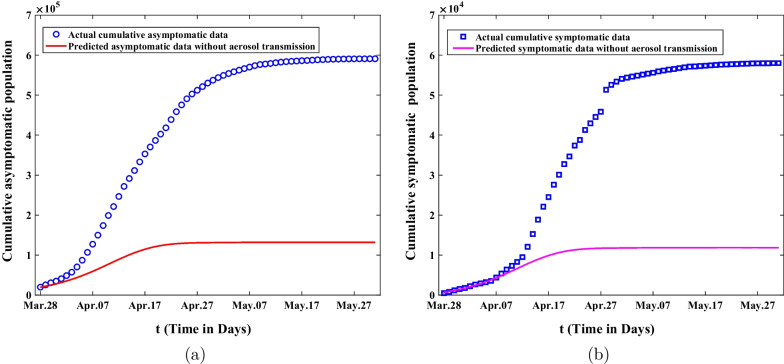
The comparisons of predicted value and actual data about the cumulative asymptomatic population and symptomatic population are showed without the aerosol transmission by agminated nucleic acid testing in the second and third stage. **a** The predicted cumulative asymptomatic population without the aerosol transmission. **b** The predicted cumulative symptomatic population without the aerosol transmission

If aerosol transmission from aggregative nucleic acid detection was not taken into account during this epidemic, accumulated asymptomatic infected individuals and symptomatic cases predicted by the model are demonstrated in Fig. 7. Figure 7a shows the comparison of predicted cumulative asymptomatic infected persons without aerosol transmission with the actual cumulative asymptomatic data. It is easy to conclude that the final size of cumulative asymptomatic persons will be reduced by about four-fifths to 130,000 on May 31. Figure 7b illustrates the comparison of predicted cumulative symptomatic cases without aerosol transmission with the actual cumulative symptomatic data. It is not hard to find that the final size of cumulative symptomatic cases will be reduced from about 58,000 to 11,730 on May 31. The eventual duration of epidemics will be much shortened.

### The spread of Omicron without additional shelter hospitals in the second stage

**Fig. 8:**
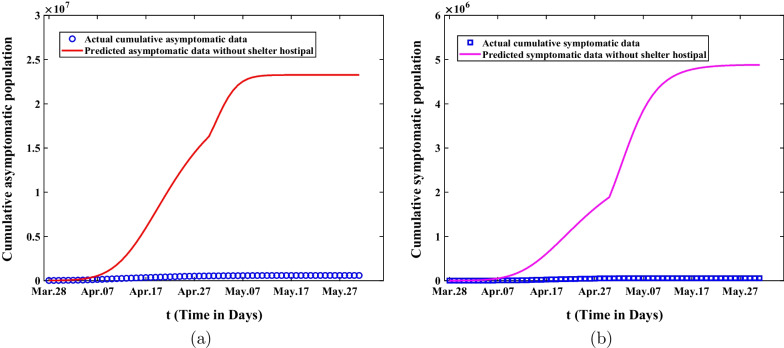
The comparisons of predicted value and actual data about the cumulative asymptomatic and symptomatic population are demonstrated without additional shelter hospitals in the second and third stages. **a** The predicted cumulative asymptomatic population without additional shelter hospitals in the second and third stages. **b** The predicted cumulative symptomatic population without additional shelter hospitals in the second and third stages

As the number of infectives increased, medical resources have been stretched and there were not enough shelter hospital beds to quarantine the asymptomatic infected people. If the government took measures to lie down and did not expand the number of shelter hospitals in the second stage, and did not take isolation measures for asymptomatic infected persons in the second and third stages, the cumulative number of infected asymptomatic and symptomatic persons predicted by the model are shown in Fig. 8. Figure 8a shows that the number of infected asymptomatic persons will increase significantly, reaching about 17 million on April 30. In the third stage, the growth rate will gradually slow down, and the final size of infected asymptomatic persons will reach 23.2 million on May 31. From Fig. 8a, we can observe that the number of infected asymptomatic people will reach 1.9 million on April 30. And then the growth rate will gradually expand in the third stage, and the final size will reach 4.88 million on May 31. According to Fig. 8, we also find an interesting phenomenon that the increase speed of asymptomatic infected persons is not synchronous with that of symptomatic infected persons. In the second stage, the increase rate of asymptomatic infected persons is relatively fast, but it gradually slow down in the third stage, while the increase rate of symptomatic infected persons is opposite. This suggests that shelter hospitals play an important role in controlling Omicron transmission.

### The spread of Omicron with adequate medical resources

**Fig. 9:**
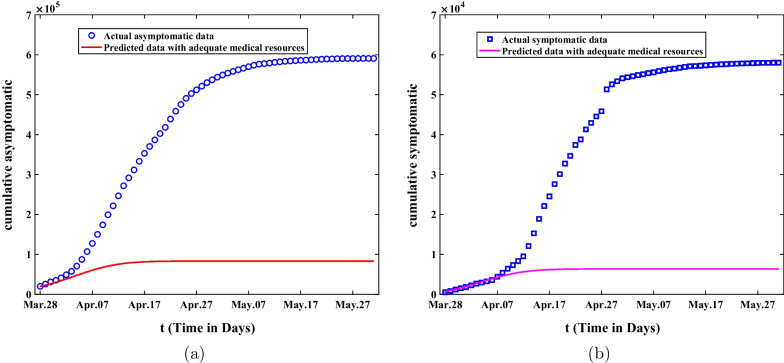
The comparisons of predicted value and actual data about the cumulative asymptomatic and symptomatic population are demonstrated under the case that if there are sufficient medical resources to cope with Omicron in the second phase. **a** The number of asymptomatic infected persons predicted by the model under adequate medical resources. **b** The number of symptomatic infected persons predicted under adequate medical resources

In this part, we will discuss the impact of adequate medical resources on the spread of Omicron in Shanghai. Here, in case, medical resources are relatively sufficient in the second stage, and there is no run on medical resources. For instance, the rate of nucleic acid testing is consistent with that of the first stage, and the number of beds in shelter hospitals can satisfy the isolation of asymptomatic persons, and the number of beds in designated hospitals can meet the normal hospitalization of symptomatic cases, the cumulative number of asymptomatic and symptomatic cases predicted by the model is shown in Fig. 9. As can be seen from Fig. 9a, b, with adequate medical resources, the number of asymptomatic infected persons would eventually shrink to 81,800, and that of symptomatic infected persons would eventually be reduced to about 6272. The duration of epidemics would be greatly shorted and the disease would be under control by the end of April.

### The spread of Omicron under different vaccinate coverage rates

**Fig. 10:**
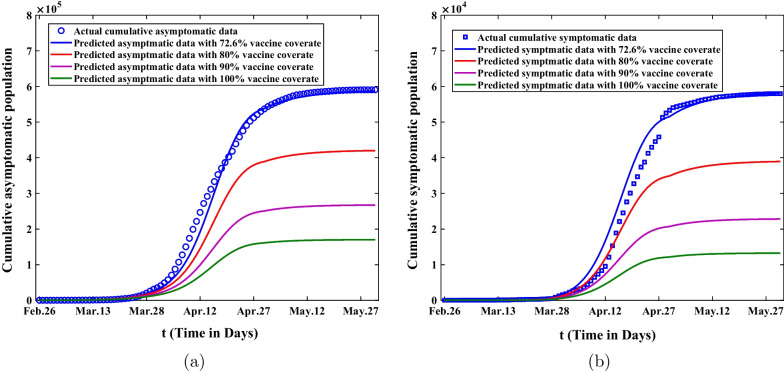
The influence of different vaccine coverage on the cumulative asymptomatic and symptomatic population. **a** The influence of different vaccine coverage on the cumulative asymptomatic population. **b** The influence of different vaccine coverage on the cumulative symptomatic population

    Here, we will investigate the influence of different vaccination coverage rates on the spread of Omicron in Shanghai. According to the literature [40], the vaccination rate of COVID-19 in Shanghai residents was about 72.6%. Figure 10 shows that as vaccination coverage increases, the cumulative number of asymptomatic and symptomatic infected persons will decrease. In other words, vaccination has a dampening effect on the spread of Omicron. Therefore, we should expand the proportion of COVID-19 vaccine as much as possible to reach the maximum. However, when the vaccine coverage rate reaches 100%, we can observe that the cumulative number of asymptomatic infected persons will be reduced to about 170,000 and the number of symptomatic infected persons will be decreased to 13,236. It also suggests that vaccination is a modest mitigation of Omicron spread, but not a complete control measure. As a result, we need to seek other strategies to block the spread of Omicron in Shanghai, such as widespread antiviral therapies, enhanced testing and strict tracking quarantine measures.

## Conclusion

Based on the specific characteristics of Omicron epidemic in Shanghai in the spring of 2022, we established a dynamic model to investigate the role of aerosol transmission caused by aggregative nucleic acid detection and the shelter hospitals in the spread of Omicron epidemic.

Firstly, we collected the daily Omicron data of asymptomatic and symptomatic infected cases in Shanghai reported by Shanghai Health Supervision Commission from February 26 to May 31, 2022 and divided the whole transmission process into three stages according to a series of control intervention measures taken by the government. We utilized the nonlinear least square method and Markov chain Monte Carlo method to estimate the parameters of the model, and the cumulative number of asymptomatic and symptomatic infected persons were used to verify the model. The numerical simulation results are shown in Figs. 3, 4, 5. It can be found that the cumulative asymptomatic individuals and symptomatic cases predicted by the model are nearly agreement with the actual reported cases.

Secondly, sensitivity analysis was applied to investigate the final size of asymptomatic and symptomatic infected persons in Shanghai under different control measures. According to Fig. 6, We can observe that the final size of asymptomatic infections will increase forty times and the final size of symptomatic cases will expand ten times without lockdown. On the other hand, it also illustrates that it is extremely necessary for the government to adopt lockdown and strict nonpharmaceutical intervention measures in Shanghai. Through the analysis of Fig. 7, we can find that the final size of cumulative asymptomatic subjects will be reduced by about four-fifths to 130,000 and the final size of cumulative symptomatic cases will be decreased to about 11,730 on May 31, 2022, and then the eventual duration of epidemics will be much shortened. Based on Fig. 8, we know that if the government takes measures to lie down and does not expand the number of shelter hospitals in the second stage, the final size of asymptomatic infected persons will reach 23.2 million and the final size of symptomatic cases will reach 4.88 million on May 31, 2022. This suggests that shelter hospitals play an important role in controlling Omicron transmission. Through discussing the impact of medical resources on the spread of Omicron in Shanghai, we discover that the number of asymptomatic infected persons will eventually shrink to 81,800, and that of symptomatic infected persons will be reduced to about 6,272 with adequate medical resources from Fig. 9. The duration of epidemics will be greatly shorted and the epidemic will be under control by the end of April. As showed in Fig. 10, the cumulative infected persons will decrease as vaccination coverage increases. Vaccination has a dampening effect on the spread of Omicron. However, vaccine doesn’t completely control the spread of COVID-19, and it is only a modest mitigation of Omicron spread. As such, we need to seek other strategies to deal with the spread of Omicron in Shanghai.

This article also admits many limitations. First, we considered that the transmission rate was the same for all people. However, researches have indicated that the effect of different age structures on the risk of infection and the influence of heterogeneity in patterns of human social contact was different [60–62]. Since Shanghai is a serious aging city, older people are more likely to be infected. Second, though the effectiveness of the vaccine varied with the duration of vaccination, the effects of vaccination we were considering were the same in this article. Third, the incidence differed widely among different districts as did the mobility of the population during the Omicron outbreak in Shanghai. Nevertheless, we didn’t take into account the spatial factors on the Omicron transmission of Shanghai. These are left for us to study in our future work.

## Data Availability

The data and materials in this study are freely available. The data of Omicron infected individuals used in this article are available on the websites(https://wsjkw.sh.gov.cn/xwfb/index.html; http://www.scdc.sh.cn/).
